# The effect of continuous positive airway pressure on obstructive sleep apnea in children with syndromic craniosynostosis

**DOI:** 10.1007/s11325-023-02981-3

**Published:** 2024-01-05

**Authors:** Yuan Chang, Yongbo Yu, Wei Zhang, Yinghui Gao, Junjun Feng, Mengjie Li, Fang Han

**Affiliations:** 1 Department of Sleep Medicine, Peking University Pepole’s Hospital, 11 Xizhimennan Road, Beijing, 100044 China; 2 Sleep Center, Peking University International Hospital, Beijing, China

**Keywords:** Obstructive sleep apnea, Syndromic craniosynostosis, Continuous positive airway pressure, Polysomnography

## Abstract

**Background:**

Obstructive sleep apnea (OSA) is common in children with syndromic craniosynostosis (SC). However, objective data on the treatment of OSA in children with SC remain inadequate. This study aimed to explore the efficacy of continuous positive airway pressure (CPAP) in the management of OSA in children with SC.

**Methods:**

A retrospective study was performed in children with SC and OSA diagnosed by polysomnography (PSG), which was defined as an apnea hypopnea index (AHI) ≥ 1. Patients were included if they were treated with CPAP and had baseline PSG and follow-up sleep studies. Clinical and demographic data were collected from all enrolled subjects.

**Results:**

A total of 45 children with SC and OSA were identified, with an average age of 6.8 ± 4.7 years. Among them, 36 cases had moderate to severe OSA (22 with severe OSA) and received CPAP therapy followed by post-treatment sleep studies. Notably, there was a significant reduction in the AHI observed after CPAP treatment (3.0 [IQR: 1.7, 4.6] versus 38.6 [IQR: 18.2, 53.3] events/h; *P* < 0.001).

**Conclusions:**

CPAP is effective and acceptable in treating severe OSA in children with SC.

## Introduction

Pediatric obstructive sleep apnea (OSA) is a common condition that affects 2% to 4% of the population [1, 2]. Recurrent episodes of upper airway obstruction during sleep lead to oxygen desaturation, hypercapnia, paradoxical thoracoabdominal motion, and sleep fragmentation [3]. Children with OSA generally suffer from several symptoms: snoring, daytime fatigue, malaise, irritability, sleep terrors, crying spells, bed wetting, delayed puberty, aggressiveness, or poor eating [4]. Left untreated, OSA may lead to long-term multiple morbidities such as cardiovascular, metabolic, cognitive and behavioral consequences [3]. Therefore, early diagnosis and treatment of OSA in children is essential to prevent long-term morbidity. Craniosynostosis is a congenital disorder associated with the premature fusion of one or more cranial sutures, which limits the normal growth of the skull, brain and face [5]. In about 40 percent of patients, craniosynostosis is part of a syndrome [6]. Syndromic craniosynostosis (SC) is associated with a range of rare genetic mutations. Depending on the particular mutation and syndrome, characteristic deformities may occur with varying severity. Craniofacial malformations and midface hypoplasia may result in reduced airway space in the nasopharyngeal and oropharyngeal airways, and during sleep, postural muscles relax, causing collapse and resulting OSA [7, 8]. In children with SC, OSA has been described since the early 1980s and is actually thought to occur in 40% to 85% of cases [9–11], significantly higher than in the general child population [12, 13].

Adenotonsillectomy is commonly recommended as a first-line treatment for OSA in the general pediatric population where adenoidal hypertrophy is a significant cause [14]. However, adenotonsillectomy is not effective in children with SC, especially in those with moderate or severe OSA [15]. Alternatively, a nasopharyngeal tube and tracheostomy are options to bypass upper airway obstruction, but the former has been associated with complications in long-term treatment, and tracheostomy is often performed in some children with severe airway obstruction from birth [16]. Midfacial advancement surgery can enlarge the nasopharynx and increase the airway dimension, which is the major cause of OSA in patients with SC [17]. Some studies have indicated that midfacial advancement surgery may help reduce the apnea hypopnea index (AHI) or respiratory disturbance index and improve OSA in SC children [18–21]. However, recent experiments have shown a significant number of children continued to have severe OSA after midfacial advancement surgery. Normalization of AHI after surgery is also rare [6, 22]. Continuous positive airway pressure (CPAP) can also be used to treat OSA in children with SC. CPAP is mostly recommended for moderate-to-severe OSA when surgical treatment failed or if patients are not considered candidates for surgical intervention [1, 14]. However, widespread use of CPAP in clinical practice is often difficult due to concerns about its effectiveness in this complex airway, as well as poor compliance and discomfort. Thus, the aim of our study was to explore retrospectively the impact of CPAP in the management of severe OSA in children with SC.

## Methods

### Patients

A retrospective chart review was conducted to identify children with SC from the maxillofacial surgery department of Peking University International Hospital who were referred to the sleep center with suspected OSA. Inclusion criteria were: children ≥ 1 and < 18 years old with SC who had overnight diagnostic polysomnography (PSG) at the sleep center between February 2015 and February 2023. Children who were < 1 year old and those who had previously had a nasopharyngeal tube, tracheostomy, CPAP treatment or maxillofacial surgery to improve upper airway obstruction were excluded. In addition, those whose first PSG utilized CPAP or titration with oxygen were excluded. All patient data were kept confidential.

### Demographic data

Demographic data including gender, date of birth, height, weight, age at time of PSG and diagnosis of SC, were obtained from the electronic medical records system. Obesity, overweight and malnutrition are defined according to the Chinese standard definition. Our study population was divided into 4 age groups and 4 body mass index (BMI) groups. The 4 age groups were: toddlers (1–3 years), preschoolers (4–6 years), school-aged children (7–12 years) and adolescents (13–17 years). The 4 BMI groups were: malnutrition (BMI < 5th percentile), normal weight (BMI ≥ 5th percentile and < 85th percentile), overweight (BMI ≥ 85th percentile < 95th percentile), and obese (≥ 95th percentile). Baseline PSG data, treatment for OSA used in each case, and follow-up sleep study data were collected.

### PSG for diagnosis OSA in SC children

The overnight PSG (Alice6, Philips Respironics Inc., United States of American) tests were performed according to the recommendations of the American Academy of Sleep Medicine (AASM) [23]. The following signals were recorded: electroencephalogram (F3M2, F4M1, C3M2, C4M1, O1M2, O2M1), bilateral electrooculogram, chin muscle electromyogram, oronasal thermistor, nasal pressure, rib cage and abdominal movement, electrocardiogram (a single modified electrocardiograph Lead II), snoring, body position, bilateral anterior tibialis electromyograms, and heart rate and oxygen saturation by pulse oximetry.

Sleep studies were scored by experienced registered polysomnographic technologists (RPSGT) using the recommended rules by the AASM Manual for the Scoring of Sleep and Associated Events: Rules, Terminology and Technical Specifications, Version 2.1 [24]. Pediatric OSA was considered present with an AHI ≥ 1. The study population was categorized into three severity groups of OSA: mild OSA (1 ≤ AHI < 5), moderate OSA (5 ≤ AHI < 10), and severe OSA (AHI ≥ 10). Normalization of the AHI after treatment was considered in those cases with AHI post-treatment < 1.

### Post-treatment sleep study

If a child with moderate to severe OSA was diagnosed with overnight PSG, we consulted with the patient and their parents and recommended CPAP therapy. For some SC children who could not wear CPAP nasal masks, we had customized special masks for them. During the CPAP titration, we estimated AHI from overnight airflow monitoring of a noninvasive ventilator (S9 Autoset, Res Med Ltd., Australia). On the same night, we simultaneously recorded heart rate and oxygen saturation using pulse oximeter (DS-5, Konica Minolta Holdings Inc., Japan). We utilized recorded estimated AHI, oxygen desaturation index (ODI), and saturation of peripheral oxygen (SpO_2_) curves to titrate the ventilator pressure. The treatment pressure level was determined as the point at which the child could tolerate it while achieving maximum reduction in AHI. The post-treatment sleep study results included the AHI, oxygen desaturation ≥ 3% index (ODI_3_), mean SpO_2_, lowest SpO_2_, time spent with SpO_2_ < 90%, and mean heart rate measured at the optimal treatment pressure level.

### Follow-up after CPAP treatment

Subsequently, we recommended long-term CPAP therapy under the titrated treatment pressure level. Children and their parents were encouraged to contact us via telephone if they encountered difficulties during CPAP therapy. Patients were offered another titration if necessary. Patients were encouraged to use CPAP therapy until they received surgical intervention or demonstrated intolerance. Additionally, follow-up telephone calls were scheduled at 1, 3, 6, 9, and 12 months post-CPAP initiation to verify the efficacy of the treatment. During the follow-up period, CPAP compliance was evaluated based on the duration of CPAP therapy usage. CPAP compliance was defined as utilizing CPAP therapy for an average of at least 4 h per night and on a minimum of 5 nights per week. Conversely, CPAP noncompliance was defined as an average daily usage of less than 4 h per day or fewer than 5 nights per week.

### Statistical analysis

Data were reported as means with standard deviations (SD) for normally distributed continuous variables, medians and interquartile ranges (IQR) for non-normally distributed continuous variables, and counts and/or percentages for categorical variables. The χ2 test was used to evaluate differences in the frequency of OSA between categories of the variables assessed such as gender, age groups, BMI groups, etc. Student t-tests were used to compare differences in the means of normally distributed continuous variables such as age, BMI and AHI, and nonparametric statistics were used to compare medians of the main polysomnographic variables assessed (total sleep time, sleep latency, rapid eye movement [REM] latency, arousal per hour of sleep, non-rapid eye movement [NREM] and REM stages, breathing events; ODI_3_, mean SpO_2_, lowest SpO_2_, etc.). A paired t-test was used to analyze the difference between pre- and post-treatment sleep study parameters. All statistical analyses were performed using SPSS 22.0 (IBM, Armonk, New York, United State of America). *P* < 0.05 was considered statistically significant.

## Results

### General data

**Fig. 1:**
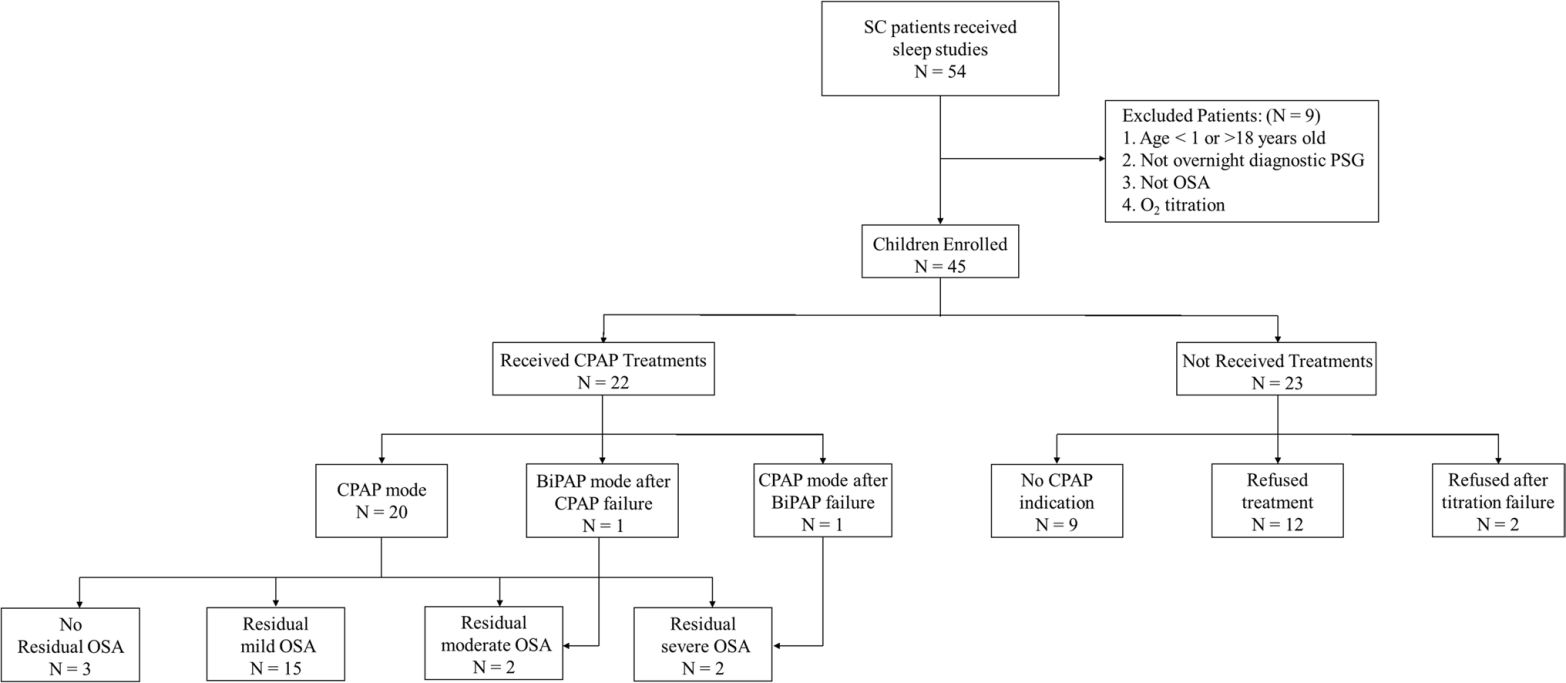
Study flowchart. *Abbreviations:* SC*,* syndromic craniosynostosis; PSG, polysomnography; CPAP, continuous positive airway pressure; OSA obstructive sleep apnea

We reviewed a total of 57 clinical records of patients with SC. Of these, 54 had undergone at least one nocturnal sleep study at our sleep center and 45 children were enrolled in our study. While some children underwent more than one PSG, for the current study only those whose first PSG was an overnight diagnostic PSG without prior treatment were included. The prevalence of OSA was 88% (45/51) in children who received diagnostic PSG. Of the 45 patients included in this study, 22 received subsequent CPAP treatment for OSA (Fig. 1). All children who received CPAP therapy had post-treatment sleep studies.

### Baseline and PSG characteristics in children with SC

The mean age of included children was 6.8 ± 4.7 y, range 1–16 y, 38% were girls. The BMI of 17 (38%) children deviated from the normal range observed in age-matched healthy Chinese controls, with 7 of them exhibiting malnutrition. The frequency of obesity was 18% (8/45) and all the obese children had severe OSA. Of the 35 children, 17 (77%) carried the fibroblast growth factor receptor (FGFR) 2 gene mutation, which is the most common mutation in Apert, Crouzon and Pfeiffer syndrome. The proportion of children with mild, moderate and severe OSA was 20 percent, 9 percent and 71 percent, respectively. Only two children were diagnosed with Apert syndrome and both had moderate OSA. Of the 45 children, 5 (11%) had Pfeiffer syndrome and 4% (2/45) had Crouzon syndrome with acanthosis nigricans. These 7 children all had severe OSA. The remaining 80% of the children had Crouzon syndrome. In children with Crouzon syndrome, 25% had mild OSA, 6% had moderate OSA, and the remaining 69% had severe OSA. Additional demographic, anthropometric, craniofacial, and data are summarized in Table 1. The resulting overnight diagnostic PSG study including sleep stages, arousals, breathing disorder events, oxygen saturation and heart rate are summarized in Table 2. The baseline AHI of all enrolled children was 29.2 ± 27.9/h.

**Table 1:** Demographic, anthropometric, craniosynostosis characteristics of syndromic craniosynostosis children with obstructive sleep apnea

Characteristic	Total Patients (*n =* 45)	Refused CPAP Therapy Patients (*n =* 14)	CPAP Treated Patients (*n =* 22)	*P* value (Refused vs. Treated)
Age※	6.8 ± 4.7	7.2 ± 3.4	6.2 ± 4.7	0.241
Toddlers Preschoolers School age Adolescents	13 (29%) 13 (29%) 13 (29%) 6 (13%)	2 (14%) 6 (43%) 6 (43%) 0 (0%)	9 (41%) 5 (23%) 4 (18%) 4 (18%)	0.142 0.273 0.140 0.141
Female	17 (38%)	5 (36%)	10 (46%)	0.732
BMI✟				
Malnutrition Normal weight Overweight Obese	7 (16%) 28 (62%) 2 (4%) 8 (18%)	2 (14%) 8 (57%) 1 (7%) 3 (21%)	5 (23%) 13 (59%) 1 (5%) 3 (14%)	0.681 > 0.999 > 0.999 0.658
Syndromic craniosynostosis				
Apert Crouzon Crouzon with acanthosis nigricans Pfeiffer	2 (4%) 36 (80%) 2 (4%) 5 (11%)	2 (14%) 11 (79%) 1 (7%) 0 (0%)	0 (0%) 16 (73%) 1 (5%) 5 (23%)	0.144 > 0.999 > 0.999 0.134
Mutant gene*	*n =* 35	*n =* 9	*n =* 19	
FGFR1 FGFR2 FGFR3 EFNB1 No Mutant	1 (3%) 27 (77%) 2 (6%) 1 (3%) 4 (9%)	0 (0%) 7 (78%) 1 (11%) 0 (0%) 1 (11%)	1 (5%) 14 (74%) 1 (5%) 1 (5%) 2 (11%)	> 0.999 > 0.999 > 0.999 > 0.999 > 0.999
OSA				
Mild	9 (20%)			
Moderate Severe	4 (9%) 32 (71%)	4 (29%) 10 (71%)	0 (0%) 22 (100%)	0.017

※The age groups: toddlers (1 to 3 years), preschoolers (4 to 6 years), school-aged children (7 to 12 years) and adolescents (13 to 17 years)

✟The BMI groups: malnutrition (BMI < 5th percentile), normal weight (BMI ≥ 5th percentile and < 85th percentile), overweight (BMI ≥ 85th percentile < 95th percentile), and obese (≥ 95th percentile)

*Abbreviations:* *BMI*, body mass index; *FGFR*, fibroblast growth factor receptor; *OSA,* obstructive sleep apnea

**Table 2:** Polysomnographic characteristics of syndromic craniosynostosis children with obstructive sleep apnea

PSG Results	Total Patients (*N* = 45)	Refused CPAP Therapy Patients (*n =* 14)	CPAP Treated Patients (*N* = 22)	*P* value (Refused vs. Treated)
Total sleep time (min) Sleep latency (min) REM latency (min) Arousal/hour sleep Sleep efficiency (%)	471.6 ± 84.4 1.5 (0.0, 7.5) 178.5 (126.3, 235.8) 15.1 (11.0, 24.8) 95.0 (89.1, 96.9)	503.6 ± 81.0 1.0 (0.0, 4.9) 178.0 (138.4, 261.5) 11.1 (14.0, 19.8) 94.1 (90.6, 98.1)	465.8 ± 74.7 3.5 (0.6, 12.0) 189.3 (145.3, 223.0) 20.0 (13.4, 29.4) 91.9 (86.1, 96.5)	0.475 0.212 0.721 0.661 0.516
Stage				
NREM 1 (%) NREM 2 (%) NREM 3 (%) REM (%)	14.5 (8.4, 21.5) 35.7 (30.8, 46.0) 29.5 (22.6, 39.7) 16.0 (10.4, 18.8)	12.0 (8.6, 16.4) 41.5 (32.9, 44.8) 27.3 (24.5, 34.6) 16.9 (12.9, 18.5)	16.3 (12.3, 27.1) 34.8 (27.5, 41.7) 32.9 (23.4, 41.8) 13.6 (5.9, 17.1)	0.284 0.123 0.256 0.127
Breathing events				
OAI (events/h) CAI (events/h) MAI (events/h) AI (events/h) HI (events/h) AHI (events/h) REM-AHI (events/h) NREM-AHI (events/h)	2.5 (0.6, 6.0) 0.6 (0.3, 1.7) 0.1 (0.0, 0.5) 4.1 (1.8, 9.2) 14.0 (3.5, 30.8) 29.2 ± 27.9 40.4 (8.7, 72.0) 15.0 (5.2, 38.3)	2.5 (1.6, 5.3) 1.0 (0.3, 2.0) 0.2 (0.0, 0.7) 4.1 (2.3, 10.5) 12.8 (7.0, 23.8) 26.3 ± 21.7 32.0 (12.3, 43.8) 11.6 (7.7, 28.9)	4.5 (2.4, 10.2) 0.7 (0.3, 1.9) 0.1 (0.0, 0.6) 6.9 (3.7, 12.3) 26.2 (13.0, 35.5) 41.9 ± 26.6 68.5 (49.8, 94.9) 35.6 (15.3, 45.3)	0.205 0.745 0.532 0.299 0.069 0.025 0.003 0.010
ODI_3_ (/h) Mean SpO_2_ (%) Min SpO_2_ (%) Time SpO_2_ < 90% (min)	17.4 (6.4, 47.5) 96.0 (92.5, 97.0) 75.0 (62.0, 88.0) 21.7 (1.1, 58.1)	17.1 (10.3, 41.6) 96.0 (94.0, 97.0) 75.0 (64.0, 83.8) 30.8 (2.7, 63.7)	32.1 (16.0, 70.5) 95.0 (88.0, 95.0) 70.0 (53.0, 75.3) 33.7 (11.0, 90.2)	0.101 0.113 0.270 0.455
Mean heart rate (/min)	91.6 ± 22.0	89.6 ± 19.2	94.3 ± 18.4	0.292

Data were reported as means with standard deviations (SD) for normally distributed continuous variables, medians and interquartile ranges (IQR) for non-normally distributed continuous variables

*Abbreviations: PSG*, polysomnography; *CPAP*, continuous positive airway pressure; *REM*, rapid eye movement; *NREM*, non-rapid eye movement; *OAI*, obstructive apnea hypopnea index; *CAI,* central apnea index; *MAI*, mixed apnea index; *AI*, apnea index; *HI*, hypopnea index; *AHI*, apnea hypopnea index; *ODI*_3_, oxygen desaturation ≥ 3% index; *SpO*_2_, saturation of peripheral oxygen

### Characteristics of SC children receiving and refusing CPAP therapy

We recommended a trial of CPAP treatment in all 36 children with SC and moderate to severe OSA. Of the 36 children 12 (4 children with moderate OSA and 8 with severe OSA) refused CPAP treatment. The pressure titration and subsequent post-treatment sleep studies were successfully performed in 22 children and 2 children withdrew because of discomfort during the titration (Fig. 1). The mean age of the 22 patients who received CPAP therapy was 6.2 ± 4.7 years, with a range of 1–16 years; among them, 46% were female. The proportions of patients with malnutrition, overweight, and obesity were 23%, 5%, and 9%, respectively. Among the patients receiving CPAP therapy, 1 patient had Crouzon syndrome with acanthosis nigricans, 5 had Pfeiffer syndrome, and the remaining 16 had Crouzon syndrome. All 22 patients suffered from severe OSA, with a baseline AHI of 41.9 ± 26.6/hour. Detailed clinical and PSG characteristics are summarized in Tables 1 and 2.

Compared to children who received CPAP therapy, the group of children who refused or withdrew from CPAP therapy (*n =* 14) exhibited a lower AHI with a mean value of 26.3 ± 21.7 events/h compared to 41.9 ± 26.6 events/h in the former group, a statistically significant difference (*P* = 0.025). (Tables 1 and 2).

### CPAP treatment in SC children with severe OSA

Among the 22 children with SC receiving CPAP therapy, 91% (20/22) achieved a therapeutic effect on CPAP mode, while 1 child required Bilevel PAP mode after a failure of CPAP. One additional child had unsatisfactory results with the application of the CPAP mode, but worse results with the Bilevel PAP mode, which was later adjusted back to the CPAP mode (Fig. 1). Even so, this patient achieved a clear reduction in AHI from CPAP therapy (from 25.6 events/h pre-treatment to 10.1 events/h post-treatment). In terms of CPAP treatment parameters, the mean pressure of CPAP mode was 10.2 ± 2.5 cmH_2_O and the IPAP and EPAP was 22/15 cmH_2_O in the Bilevel PAP mode.

**Fig. 2:**
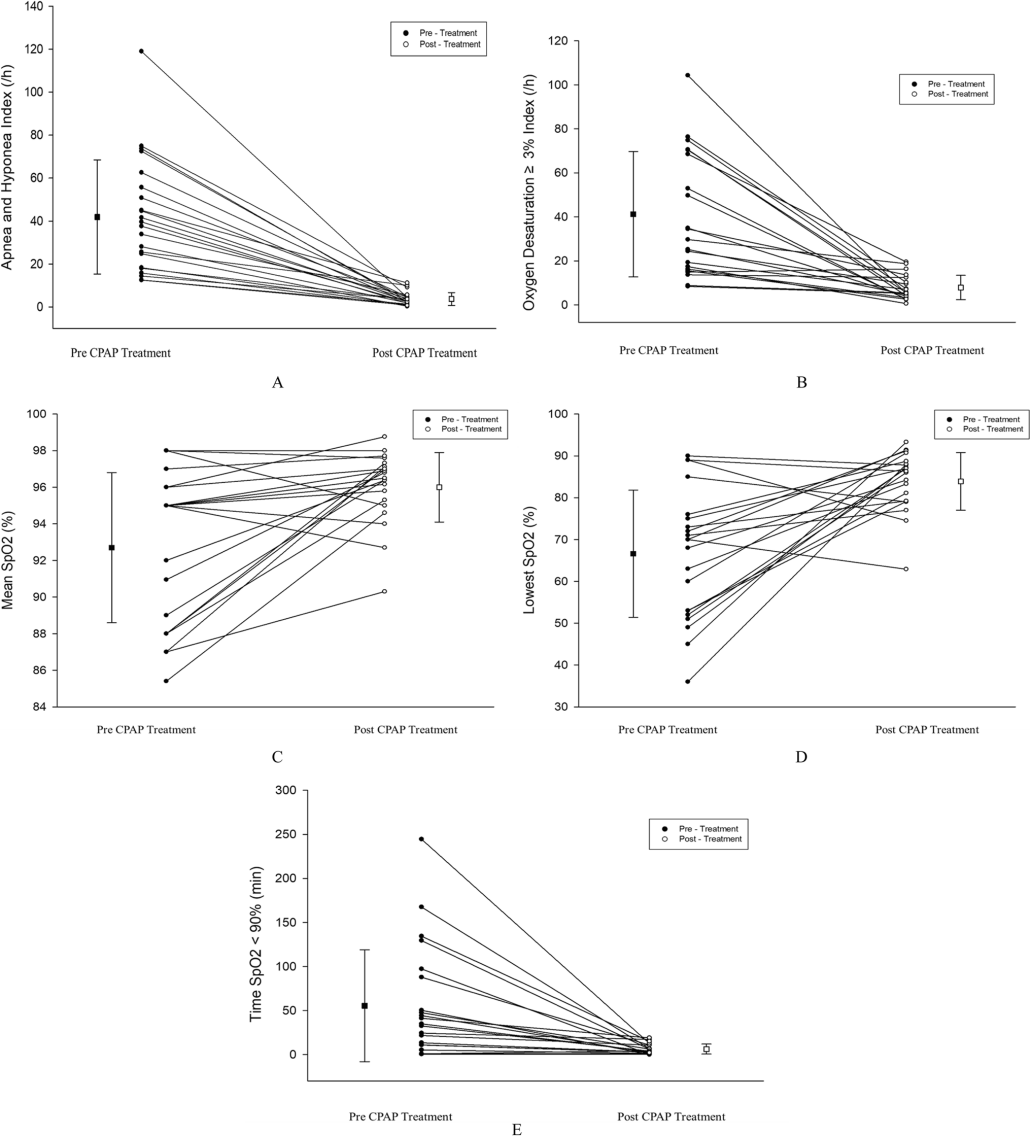
The respiratory and sleep parameters of pre CPAP treatment and post CPAP treatment in 22 patients. Apnea and hypopnea index (**A**), oxygen desaturation ≥ 3% index (**B**), mean pulse oxygen saturation (**C**), lowest pulse oxygen saturation (**D**), and time pulse oxygen saturation < 90% (E) for each individual is shown for those values pre CPAP treatment (closed circles) and post CPAP treatment (open circles), and values are connected by a line. Group values presented as mean ± standard deviation by a dash and whiskers. Differences are significant (all *P* < 0.05). *Abbreviations:* CPAP, continuous positive airway pressure; SpO2, saturation of pulse oxygen

Table 3 provides baseline and post-treatment sleep study measurements for the CPAP treatment. A significant reduction in AHI was found (from 41.9 ± 26.6 events/h to 3.4 ± 2.7 events/h, *P* < 0.001), as well as a reduction in ODI_3_ (from 32.1 [IQR: 16.0, 70.5]/h to 5.5 [IQR: 3.7, 5.5]/h, *p* < 0.001) after treatment. There were significant improvements in the mean SpO_2_, lowest SpO_2_ and time of SpO_2_ < 90%. No significant difference in mean heart rate was found after CPAP treatment (94.3 ± 18.4 versus 92.3 ± 14.3, *P* = 0.371). The AHI, ODI_3_, mean SpO_2_, lowest SpO_2_, and time SpO_2_ < 90% of pre- and post- CPAP treatment collected are shown in Fig. 2. Among the children who received CPAP treatment, 3 (14%) resolved on post-treatment sleep study; 15 (68%) improved to mild OSA, 2 (9%) had moderate OSA and 2 (9.1%) still suffered with severe OSA (Fig. 1).

**Table 3:** Baseline polysomnography and post-treatment sleep study values on CPAP treatment

Sleep test results	CPAP treatment (*n =* 22)		*P* value
	Baseline	Post-treatment	
AHI (events/h)	41.9 ± 26.6	3.4 ± 2.7	< 0.001
ODI_3_ (/h)	32.1 (16.0, 70.5)	5.5 (3.7, 5.5)	< 0.001
Mean SpO_2_ (%)	95.0 (88.0, 95.0)	96.4 (95.0, 97.0)	0.002
Lowest SpO_2_ (%)	70.0 (53.0, 75.3)	86.2 (79.1, 88.1)	0.001
Time SpO_2_ < 90% (min)	33.7 (11.0, 90.2)	4.2 (1.9, 10.8)	< 0.001
Mean heart rate (/min)	94.3 ± 18.4	92.3 ± 14.3	0.371

Data were reported as means with standard deviations (SD) for normally distributed continuous variables, medians and interquartile ranges (IQR) for non-normally distributed continuous variables

*Abbreviations: CPAP*, continuous positive airway pressure; *AHI*, apnea hypopnea index; *ODI*_3_, oxygen desaturation ≥ 3% index; *SpO*_2_, saturation of peripheral oxygen

### CPAP compliance of SC children receiving CPAP therapy

In subsequent telephone follow-up, the duration of CPAP therapy for SC children who received CPAP was 30.0 (IQR: 10.3, 76.5) days, range 5–180 days. Of these, 13/22 (59%) children belonged to the CPAP compliance group, with a treatment duration of 30.0 (IQR: 8.0, 50.0) days. The remaining 9/22 (41%) constituted the CPAP noncompliance group, who underwent CPAP therapy for a period of 30.0 (IQR: 14.0, 82.0) days. There were no significant differences observed in age, gender, BMI, AHI, and mean SpO_2_ between the two groups (all *P* > 0.05). More children in the CPAP compliance group discontinued CPAP therapy due to surgical intervention, while a higher number of patients in the CPAP noncompliance group ceased therapy because of discomfort or perceived lack of efficacy (*P* = 0.007). Furthermore, there appeared to be a relatively lower post-treatment AHI in the CPAP compliance group compared to the noncompliance group though this difference did not achieve statistical significance (Table 4).

**Table 4:** CPAP compliance of children with syndromic craniosynostosis receiving CPAP therapy

Characteristic	All CPAP Treated Patients (*N* = 22)	CPAP Compliant (*n =* 13)	CPAP Noncompliant (*n =* 9)	*P* value
Age	6.2 ± 4.7	5.8 ± 3.8	6.9 ± 5.9	0.948
Female	9	5	4	> 0.999
BMI	17.8 ± 5.4	`17.9 ± 5.6	17.7 ± 5.5	0.845
Syndromic craniosynostosis				
Crouzon Crouzon with acanthosis nigricans Pfeiffer	16 1 5	8 1 4	8 0 1	0.333 > 0.999 0.360
Treatment pressure level	10.2 ± 2.5	10.3 ± 2.5	9.9 ± 2.6	0.702
Pre-treatment AHI	41.9 ± 26.6	42.9 ± 32.6	41.6 ± 18.8	0.695
Post-treatment AHI	3.4 ± 2.7	3.1 ± 2.8	4.6 ± 3.2	0.164
No residual Residual mild OSA Residual moderate OSA Residual severe OSA	3 15 2 2	2 10 0 1	1 5 2 1	> 0.999 0.376 0.156 > 0.999
Pre-treatment mean SpO_2_	95.0 (88.0, 95.0)	95.0 (91.0, 96.0)	92.0 (87.0, 95.0)	0.209
Post-treatment mean SpO_2_	96.4 (95.0, 97.0)	96.8 (95.8, 97.3)	96.4 (94.6, 97.0)	0.393
CPAP therapy duration	30.0 (10.3, 76.5)	30.0 (8.0, 50.0)	30.0 (14.0, 82.0)	0.431
Cause of CPAP termination				
Surgery Discomfort or Inefficiency	13 9	11 2	2 7	0.007

Data were reported as means with standard deviations (SD) for normally distributed continuous variables, medians and interquartile ranges (IQR) for non-normally distributed continuous variables

*Abbreviations: CPAP*, continuous positive airway pressure; *BMI*, body mass index; *OSA*, obstructive sleep apnea; *AHI*, apnea hypopnea index; *ODI*_3_, oxygen desaturation ≥ 3% index; *SpO*_2_, saturation of peripheral oxygen

## Discussion

Our retrospective review showed that the frequency of OSA in children with obvious midface hypoplasia referred to sleep clinics was approximately 90%, and the majority of these children had severe OSA. This is higher than reported in previous studies, which ranged from 40 to 83% [9–11]. However, in previous studies, the prevalence of OSA was reported for all kinds of children with SC. Our study involved only children with Apert, Crouzon, Crouzon with acanthosis nigricans and Pfeiffer syndrome who had obvious midface hypoplasia and all OSA was diagnosed only by the first overnight PSG. Packer et al. also reported a higher prevalence of OSA in Apert, Crouzon, and Pfeiffer syndrome than in Muenke and Saethre-Chotzen syndrome [25]. Hence, the high prevalence in the current study is easily explained.

Treatment of OSA in children with SC is often different than in children without SC. Relief of nasopharyngeal and oropharyngeal obstruction appears to be considered as a good treatment modality for compromised OSA in children with SC. Respiratory support through noninvasive treatment with CPAP can overcome the obstruction. In 1996, Gonsalez et al. reported on the effectiveness of CPAP in the treatment of OSA in children with SC. CPAP was used successfully in 5 of the 8 children [26]. However, lack of fitting nasofacial masks for children with facial deformities and the complicated application of CPAP in children with anatomic narrowing nasopharynx are real world complications [16]. These concerns have further reduced the use of CPAP in clinics.

The results of our study suggest that CPAP therapy may be an acceptable and effective treatment for children with SC and severe OSA. The follow-up study demonstrated that establishing a clear treatment goal and timeframe for surgical intervention in children receiving CPAP therapy proved advantageous in enhancing CPAP compliance. However, the children in our study used CPAP for a short period. Most patients used CPAP only as a bridging therapy prior to surgery. Other studies have shown that long-term CPAP use in children with OSA have poor adherence to therapy. Also the wear of a CPAP mask may cause craniofacial dysplasia [1, 27]. Therefore, longer term studies are needed to explore the feasibility of CPAP treatment in children with SC.

However, normalization of AHI after surgery treatment is rare [6, 22] and the timing of the midface advancement procedure is currently controversial. Some medical centers believe that the minimum age for surgery may be advanced to 1–2 years of age if there is severe upper airway obstruction, such as severe OSA or exophthalmos, and 6–8 years of age is recommended for corrective surgery if there is no severe airway obstruction [28]. There are also specialists who advocate waiting for the children's bones to mature before performing the surgery [29, 30]. Specialists who advocate early correction believe it can improve the lives of children with alopecia areata, even if a second operation is required later. Specialists who advocate for delayed surgery argue that the incidence of malocclusion is high in children who undergo early surgery, and that patients often need to be corrected by surgery again in their teens [31]. Corrective surgery of craniosynostosis in childhood is difficult to perform and carries extreme risks. A big problems in the surgical correction of craniosynostosis is the occasional massive blood loss (20% to 500% of the circulating volume), which may occur in a relatively short period and in patients with age-related circulating volume [32]. Several studies have suggested that age < 18 years may predict large blood loss [33, 34]. Our study offers a justification for utilizing CPAP therapy as a bridging treatment during the preoperative period for children with SC and severe OSA allowing the patient to choose the best time for midface advancement procedure, and perhaps even to avoid secondary surgery.

An important limitation of the current study is that all children received CPAP treatment and did not undergo a follow-up PSG. Most of the children in our study were very young. Parents often find it unacceptable for their children to wear complex testing equipment during CPAP therapy. Fortunately, the AHI estimated by the noninvasive ventilator matches the ODI measured by the pulse oximeter [35]. These two simultaneous improvements of the two devices can validate the treatment effect of CPAP to a certain extent. There are other limitations to our study. This is a retrospective observational study and is limited by the available data suggesting the possibility of bias. Access to the data was limited to the medical records of children treated at our hospitals during those years limiting the generalizability of the results. No prior power analysis was performed to determine the required number of patients to avoid a beta error. In addition, to eliminate confounding factors, a rigorous chart review was performed to ensure that patients met all inclusion criteria. However, excluding patients may inadvertently lead to new selection biases. Thus, future multicenter prospective studies with larger patient cohorts are necessary to confirm our results and to control for these confounding factors.

This study was an investigation into the effect of CPAP therapy in children with SC and OSA. Based on PSG parameters and other sleep studies, the study suggests that CPAP treatment may be effective and acceptable in treating severe OSA in children with SC. 

## Data Availability

Data will be made available on reasonable request.

## Declarations

This was a retrospective study and all patient data were kept strictly confidential.
